# CoeViz: a web-based tool for coevolution analysis of protein residues

**DOI:** 10.1186/s12859-016-0975-z

**Published:** 2016-03-08

**Authors:** Frazier N. Baker, Aleksey Porollo

**Affiliations:** Department of Electrical Engineering and Computing Systems, University of Cincinnati, 2901 Woodside Drive, Cincinnati, OH 45221 USA; Center for Autoimmune Genomics and Etiology, Cincinnati Children’s Hospital Medical Center, 3333 Burnet Avenue, Cincinnati, OH 45229 USA; Division of Biomedical Informatics, Cincinnati Children’s Hospital Medical Center, 3333 Burnet Avenue, Cincinnati, OH 45229 USA

**Keywords:** Coevolution, Coevolution analysis, Coevolving residues, Co-occurring residues, Covariation of residues, Protein structure, Protein function, Protein annotation, Web-server

## Abstract

**Background:**

Proteins generally perform their function in a folded state. Residues forming an active site, whether it is a catalytic center or interaction interface, are frequently distant in a protein sequence. Hence, traditional sequence-based prediction methods focusing on a single residue (or a short window of residues) at a time may have difficulties in identifying and clustering the residues constituting a functional site, especially when a protein has multiple functions. Evolutionary information encoded in multiple sequence alignments is known to greatly improve sequence-based predictions. Identification of coevolving residues further advances the protein structure and function annotation by revealing cooperative pairs and higher order groupings of residues.

**Results:**

We present a new web-based tool (CoeViz) that provides a versatile analysis and visualization of pairwise coevolution of amino acid residues. The tool computes three covariance metrics: mutual information, chi-square statistic, Pearson correlation, and one conservation metric: joint Shannon entropy. Implemented adjustments of covariance scores include phylogeny correction, corrections for sequence dissimilarity and alignment gaps, and the average product correction. Visualization of residue relationships is enhanced by hierarchical cluster trees, heat maps, circular diagrams, and the residue highlighting in protein sequence and 3D structure. Unlike other existing tools, CoeViz is not limited to analyzing conserved domains or protein families and can process long, unstructured and multi-domain proteins thousands of residues long. Two examples are provided to illustrate the use of the tool for identification of residues (1) involved in enzymatic function, (2) forming short linear functional motifs, and (3) constituting a structural domain.

**Conclusions:**

CoeViz represents a practical resource for a quick sequence-based protein annotation for molecular biologists, e.g., for identifying putative functional clusters of residues and structural domains. CoeViz also can serve computational biologists as a resource of coevolution matrices, e.g., for developing machine learning-based prediction models. The presented tool is integrated in the POLYVIEW-2D server (http://polyview.cchmc.org/) and available from resulting pages of POLYVIEW-2D.

## Background

Protein folding and function are determined by groups of amino acid residues, which are usually located distantly in the sequence but tend to appear in spatial proximity. Sequence-based identification of residues critical in protein structure or function is a long standing problem in structural bioinformatics. On the other hand, demand for sequence-based annotations has been increasing in the age of modern high-throughput genome and transcriptome sequencing.

Both protein structure and functional site prediction methods utilize evolutionary information derived from a multiple sequence alignment (MSA) usually with the focus on individual residues. At the same time, cooperative nature of protein folding and function determined by groups of residues distant in sequence prompted many studies for identification of coevolving residues from the MSA. Earlier methods identified correlated mutations using mutual information [1, 2], Pearson correlation coefficient (also known as McBASC) [3–6], χ^2^ statistic (also known as OMES) [7], and two-state maximum likelihood [8]. An alternative approach was to express amino acid covariance using a statistical coupling energy (∆∆*G*) defined as the difference in “free energy” between the full sequence alignment and subalignment (also known as statistical coupling analysis, SCA) [9], which was later updated to simplify the definition of ∆∆*G* [10, 11]. The more recent advanced methods utilize approaches from statistical physics to discriminate direct and indirect correlations (direct-coupling analysis, DCA) [12, 13], with further improvements by introducing the inverse Potts model algorithm and a pseudolikelihood maximization procedure (plmDCA) [14]. Another recent method, PSICOV, employs sparse inverse covariance estimation to identify true covariation signal in the MSA [15].

Sequence databases that are used to generate MSA may present considerable overrepresentation of some species compared to others, a human-introduced bias driven by research interests. Therefore, many sequences may be derived from closely related species that did not have time to diverge to represent truly independent sequences from the same protein family. This effect is called phylogenetic noise or bias. One of the major challenges in coevolution analysis is to reduce this noise from the MSA. Earlier approaches were to weigh contribution of each aligned sequence by its sequence identity to a query protein or by the number of gaps in the alignment. Modern methods introduce a separate procedure to account for phylogenetic bias in the MSA mitigating the influence of the multiple closely related sequences (see, e.g., MirrorTree [16], CAPS [17], DCA [13], PSICOV [15]). These procedures are estimated to take most of the computational time in the overall coevolution analysis [18]. An alternative fast approach for improving mutual information without considering explicitly the phylogeny in the MSA was suggested by adjusting the covariance metric with the average product correction (APC) [19].

Recent successful examples of utilizing the coevolving residues include predictions of inter- and intra-protein residue-residue contacts [20–22], and prediction of mutation effects [23]. Further reading on the methods for identification of coevolving residues in proteins and their various applications can be found in recent reviews [18, 24]. Collectively, with all apparent advantages of methods in coevolution analysis that greatly facilitate protein modeling and functional annotations, there are certain limitations impeding biologists to widely utilize these methods, including requirements for considerable computational resources and restrains to relatively short proteins or conserved domains.

CoeViz was developed to provide molecular biologists with a web-based tool that can deal with proteins thousands of residues long enabling a fast, automated, and interactive analysis of coevolution data derived using a variety of covariance metrics and different corrections. The tool provides versatile means to identify and visualize inter-residue contacts and groups of residues involved in the same function. Two examples are presented to illustrate identification of the residues constituting (1) a catalytic site in Cys-Gly metallodipeptidase (SwissProt: DUG1_YEAST), and (2) functional linear motifs and repeats in the APC/C activator protein Cdc20 (SwissProt: CDC20_YEAST).

### Implementation

#### Coevolution and conservation metrics

Unless the MSA for a given protein is provided by the user, alignments are generated on the server side using three iterations of PSI-BLAST [25] with the profile-inclusion threshold of expect (e)-value = 0.001 and the number of aligned sequences 2000. The sequence homology search can be done against the Pfam [26] or NCBI NR databases. The latter database is represented by three options: full and reduced to 90 % or 70 % sequence identity by CD-HIT [27]. While PSI-BLAST generates local alignments, coevolution metrics are still computed from them because (1) refinement by global alignments can be very computationally intensive for thousands of sequences; (2) global alignment algorithms may fail for multi-domain proteins (especially those homologs with an alternative order of the domains); and (3) local alignments are sufficient for coevolution analysis as illustrated in [13].

Coevolution scores are computed from the MSA using three different covariance metrics: mutual information (*MI*, Eq. 1) [2], chi-square statistic (χ^2^, Eq. 2) [7], and Pearson correlation (*r*, Eq. 3). Conservation is defined by the joint Shannon entropy (*S*, Eq. 4). Each metric, in turn, is computed using four weighting schemes: weighted by sequence dissimilarity or sequence gapping in the alignment (Eqs. 5 and 6), by phylogeny background as defined in [13] (Eq. 7), and non-weighted. *MI* scores have an additional adjustment using the average product correction (*APC*, Eq. 8) to produce *MIp* scores (Eq. 9) [19]. All metrics based on frequencies are computed using four states as possible combinations of amino acids at two positions (*i* and *j*), where each amino acid is either equal (X) or not equal (!X) to the one in the query sequence.

1 $$MI\left(i,j\right)={\displaystyle {\sum}_x{\displaystyle {\sum}_y{p}_{ij}\left(x,y\right) \log \frac{p_{ij}\left(x,y\right)}{p_{i}(x){p}_j(y)}}}$$

2 $${x}^2\left(i,j\right)={\displaystyle {\sum}_x}{\displaystyle {\sum}_y}\frac{{\left({p}_{ij}\left(x,y\right)-{p}_i(x){p}_j(y)\right)}^2}{p{}_i(x){p}_j(y)}$$

3 $$r\left(i,j\right)=\frac{1}{N_{eff}}{\displaystyle {\sum}_l\frac{w_{sl}\left({s}_{il}-{\overline{s}}_l\right)\left({s}_{jl}-{\overline{s}}_j\right)}{\sigma_i{\sigma}_j}}$$

4 $$S\left(i,j\right)=-{\displaystyle {\sum}_x{\displaystyle {\sum}_y{p}_{ij}\left(x,y\right) \log {p}_{ij}\left(x,y\right)}}$$

5 $$p(s)=\frac{w_{sl}}{N_{eff}+\lambda }$$

6 $${N}_{eff}={\displaystyle {\sum}_l{w}_{sl}}$$

7 $${w}_a^{ph}=1/\left|\left\{b\in \left\{1,\dots, N\right\}\left| seqid\left({A}^a,{A}^b\right)>80\%\right.\right\}\right|$$

where *x* = {X; !X} and *y* = {Y; !Y}; *p* (*s*) is the observed frequency of state *s* = {x; y; x,y}; *N*_*eff*_ is the effective sum of weights of alignments where both positions are not gaps. *w*_*sl*_ is a weighted count of state *s*, which is equal to 1 for non-weighted scores, 1–(percent of sequence identity) or 1–(percent of gaps) of the alignment *l* for weighting by sequence dissimilarity or alignment gapping, respectively, and *w*_*a*_^*ph*^ for weighting by phylogeny. *w*_*a*_^*ph*^ is a weight for sequence *A*^*a*^ in the MSA of *N* total sequences that equals to one over the number of sequences *A*^*b*^ in the MSA that have at least 80 % sequence identity to *A*^*a*^. 80 % was chosen as a midpoint of the range 70–90 %, where there is no strong dependence observed on the precise threshold value [13]. *s*_*il*_ is a similarity score that quantifies the change of an amino acid at position *i* to the one in the aligned sequence *l*. ${\overline{s}}_l$ and σ_*i*_ are mean and standard deviation, respectively, of all similarity scores of changes for a given position represented across the all sequences aligned to the query. Similarity scores are taken from the position specific similarity matrix (PSSM) generated by PSI-BLAST. λ is a pseudo count, which is equal to 1 for all metrics here.

8 $$APC\left(a,b\right)=\frac{MI\left(a,\overline{x}\right)MI\left(b,\overline{x}\right)}{\overline{MI}}$$

9 $$MIp\left(a,b\right)=MI\left(a,b\right)-APC\left(a,b\right)$$

where $MI\left(a,\overline{x}\right)$ is the mean *MI* of column *a*, and $\overline{MI}$ is the overall mean *MI*.

Negative values of *MIp* scores are assigned to 0, and then all *MI* scores are min-max normalized to range [0, 1]. *S* is normalized to the same range by factor 1/log (4). χ^2^ values are converted to the corresponding cumulative probabilities at degree of freedom (df) = 1.

Scores for each metric are organized in symmetrical matrices with the main diagonal presenting plain or weighted frequencies, as defined above, of each individual residue for *MI*-and χ^2^-based metrics, and the individual Shannon entropies using 20 states (20 amino acids) for *S*-based metric. Individual entropies are computed using probability part of the PSSM files from the PSI-BLAST output and normalized to range [0, 1] by factor 1/log (20). Residues of the query protein are clustered using hierarchical clustering with the complete linkage method. Prior to clustering, negative *r* scores are assigned to 0; *MI*, *r*, and χ^2^ scores are converted to distances by 1–score transformation. Both the clustering and conversion of χ^2^ to cumulative probabilities are performed using the R statistical package (functions hclust and pchisq, respectively).

### Web Interface

**Fig. 1:**
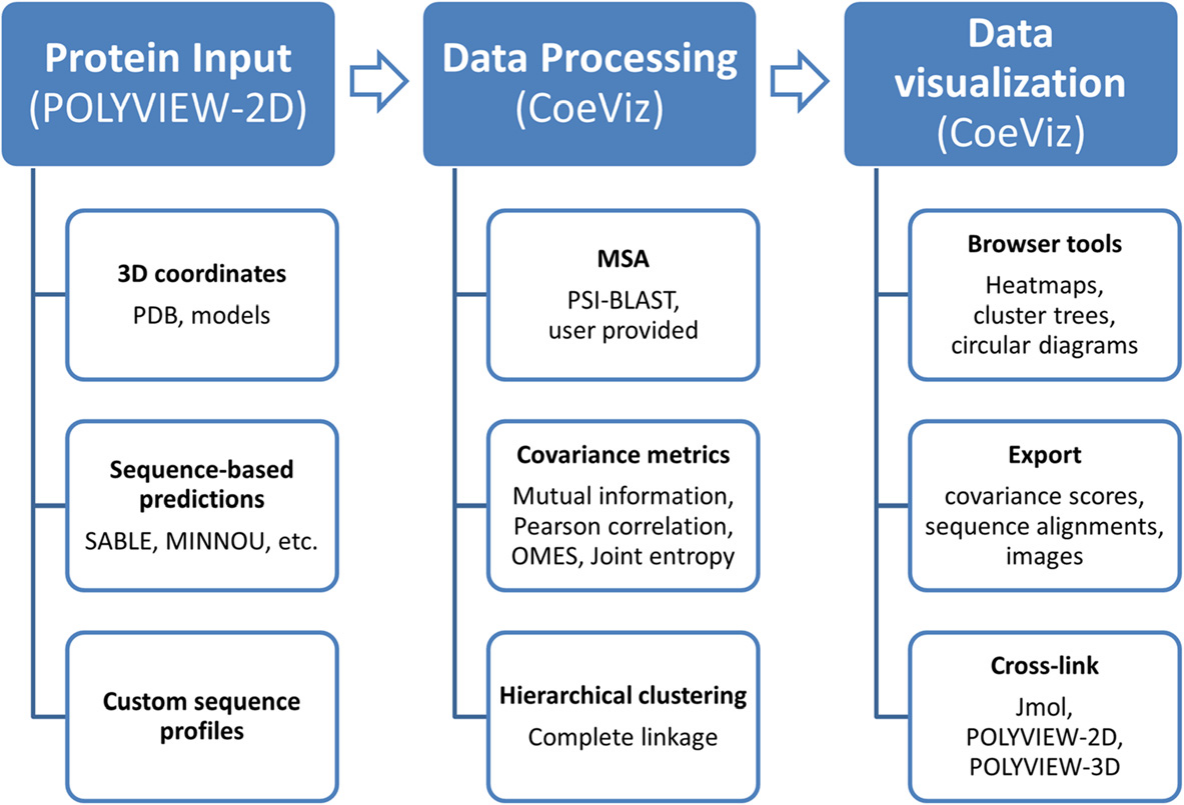
A flowchart of CoeViz. Protein data are submitted as defined in the POLYVIEW-2D server ([28], http://polyview.cchmc.org/polyview_doc.html), which includes PDB-formatted coordinate files, output from the sequence-based prediction servers, or custom sequence profiles. At the protein visualization page, there is an option provided to request analysis of covariance of amino acids (CoeViz). The user can choose a covariance metric and a database to generate the MSA or provide a file with the constructed MSA. CoeViz computes a requested covariance or conservation metric with all implemented adjustments separately and performs hierarchical clustering. Once calculations are completed, CoeViz provides an interactive web-interface to review covariance data using heatmaps, circular diagrams, and clustering trees. From the circular diagrams, the user has options to map identified correlated amino acids to a protein 3D structure or sequence depending on the input data. All generated results can be exported in text or graphics formats

The web interface for coevolution analysis (CoeViz) is implemented as part of the protein visualization server POLYVIEW-2D [28] that shows CoeViz as an option for the further sequence-based analysis from its resulting pages (Fig. 1). CoeViz accounts for a custom residue numeration (e.g., non-consecutive or with insertion codes), which is common for proteins deposited in Protein Databank (PDB, [29]). A request for analysis initiates MSA and coevolution calculations on the server side that may take from minutes to hours depending on the query sequence length, size of the generated MSA, and load of the computing cluster. Once all scores for a requested metric with different weighting schemes are computed, the subsequent analysis, visualization, and switching between the adjustments for the given metric are conducted in real time.

The computed data can be interactively explored using heat maps at different zoom levels. The color gradient is from blue (0 = no covariation) through white (0.5 = moderate covariance) to red (1 = complete covariance) for *MI*-, *r*-, and χ^2^-based metrics, whereas for joint entropy it is blue (1 = no joint conservation) through white to red (0 = complete joint conservation). Cluster trees are static; however, the cluster tree image is automatically updated when a different adjusted metric is chosen. In addition to residue labeling, the cluster tree leaves are colored according to hydropathic properties of amino acids, which may facilitate identification of clusters of hydrophobic or charged residues. The color convention follows the previous definition in POLYVIEW-2D and can be found on its documentation web-page. Residue groupings can also be reviewed through interactive circular diagrams. These diagrams allow for navigation based on residue relationships, rather than on position within the sequence. Once a set of related residues is defined on the diagram, they can be automatically mapped to the protein 3D structure using the Jmol applet [30] or POLYVIEW-3D server [31] if the input to POLYVIEW-2D was a protein coordinate file (e.g., from PDB). Otherwise, they can only be mapped to a protein sequence using POLYVIEW-2D [28].

The interactive web interface utilizes D3 [32] and Aight (https://github.com/shawnbot/aight) JavaScript libraries. Data export options include images of cluster trees (in the PNG format), a current view of the heat map (PNG), and relational circular diagrams (SVG). All generated matrices with coevolution scores, as well as the underlying MSA, can be exported in tab-separated text format.

## Results and discussion

**Fig. 2:**
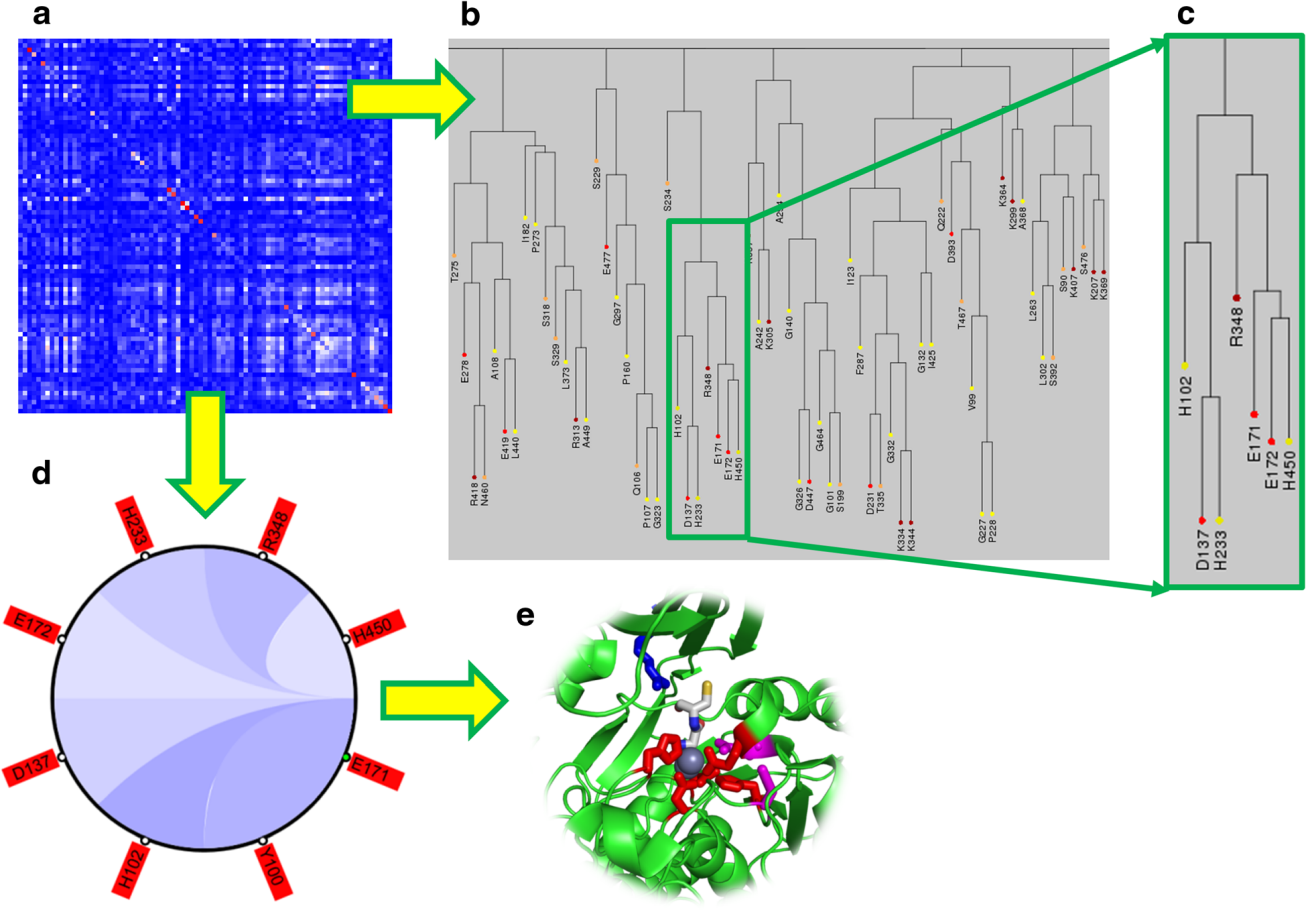
Amino acid coevolution profile reveals residues constituting the active site of the Cys-Gly metallodipeptidase (SwissProt: DUG1_YEAST). **a** A fragment of the heat map displaying amino acid coevolution computed using χ^2^ weighted by sequence dissimilarity derived from sequence alignments to the protein sequence defined in PDB ID 4G1P against NR database with 90 % identity reduction. **b** A fragment of the cluster tree derived from the chi-square data converted to a distance matrix. **c** The zoomed in cluster of amino acids that contains known Zn binding residues (H102, D137, E172, H450) and a catalytic site (E171). **d** From the heat map, one can retrieve a circular diagram representing the closest relationships to a given residue; here is to the one of catalytic residues (E171) after applying a ≥0.3 cutoff to χ^2^-based cumulative probabilities. **e** From the circular diagram, one can map the clustered residues to the submitted protein 3D structure; here is to DUG1 (PDB:4G1P). Residues highlighted red (H102, D137, E172, D200, H450) are amino acids binding Zn (grey spheres); magenta – catalytic residues (D104, E171); blue is a residue involved in substrate recognition (R348). The substrate (Cys-Gly) is rendered as sticks colored by an atom type

Figure 2 illustrates how CoeViz can help identify functionally important residues using a peptidase from baker’s yeast (SwissProt: DUG1_YEAST) as an example. Dug1p is a Cys-Gly dipeptidase and belongs to the M20A family of metallopeptidases [33]. The enzyme requires two Zinc ions in the active site to cleave the substrate. Based on χ^2^ scores weighted by sequence dissimilarity, residues binding Zn (H102, D137, E172, H450) and a catalytic residue (E171) are clustered together (Fig. 2b). Interestingly, R348 is in the same cluster (Fig. 2c). When the residues are mapped to 3D structure available in Protein Databank (PDB:4G1P), where the enzyme is co-crystallized with the substrate and Zn ions in the active site, R348 appears to be on the opposite side of the active site cavity and in contact with the substrate (Fig. 2e) suggesting its role in substrate recognition and positioning the dipeptide into the catalytic center. On the other hand, when the closest relationships are reviewed for residue E171, all the functional residues, Zn binding and catalytic, appear on the diagram (Fig. 2d).

The same protein structure was submitted to the ConSurf server [34] to see if it can identify the catalytic site. Out of 480 residues, 150 were found to be highly conserved (score 9), majority of which are in a protein core and most likely involved in protein folding, not function. These results illustrate the limits of the single residue conservation based methods in identification of functional sites, when they cannot distinguish functionally important residues from the structural determinants.

**Fig. 3:**
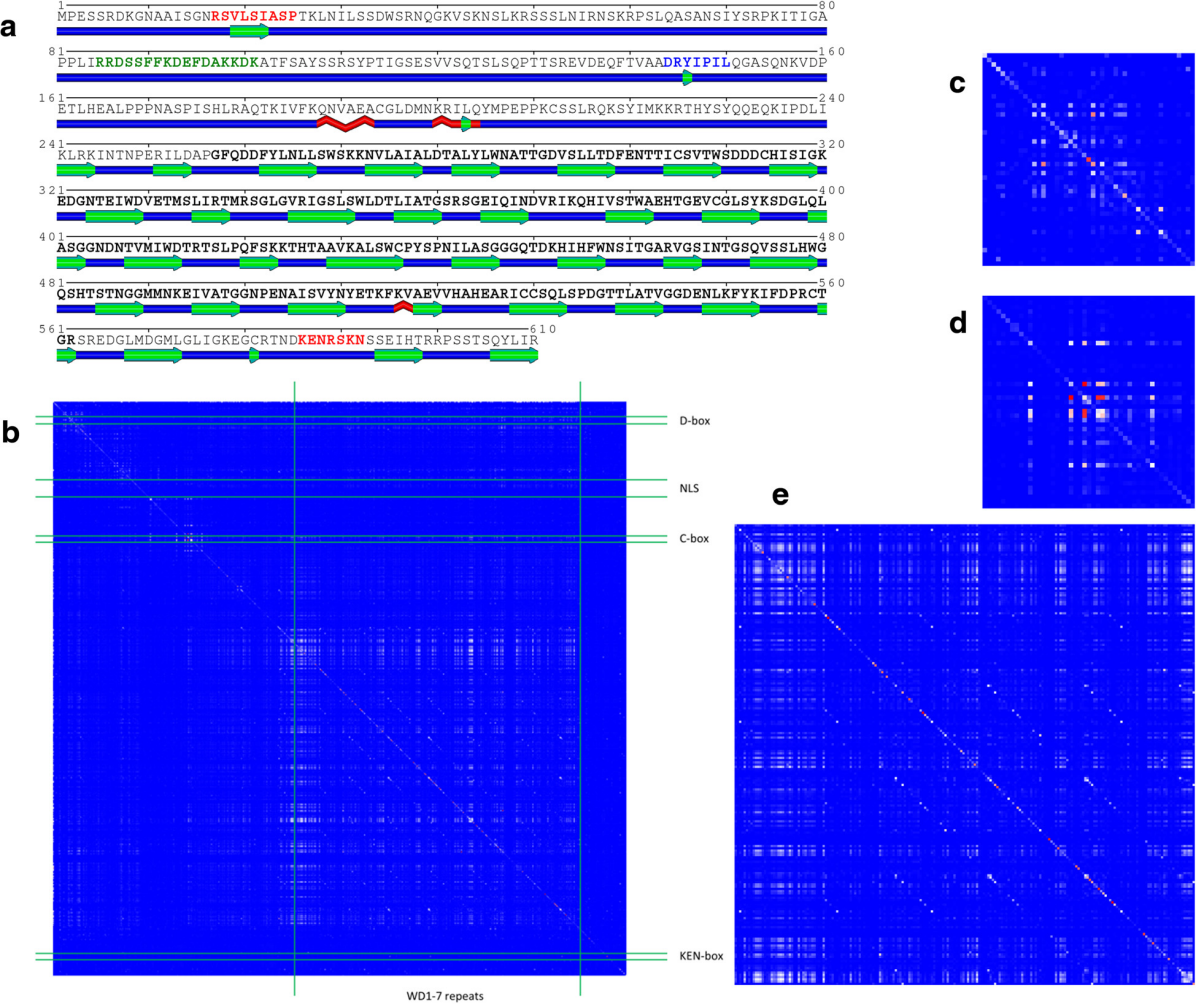
Amino acid coevolution profile reveals residues constituting a structural domain and locations of the functional linear motifs in Cdc20 (SwissProt: CDC20_YEAST). **a** SS prediction by SABLE visualized by POLYVIEW-2D with residues highlighted in functional motifs and a structural domain: red – residues constituting D- and KEN-boxes; green–residues in the bipartite NLS; blue–C-box; residues with bold face are in the WD-repeats domain. Keys for graphical SS elements can be found in the POLYVIEW-2D documentation. **b** A full heat map displaying amino acid coevolution computed using *MI* weighted by phylogeny and derived from sequence alignments to the protein sequence defined in UniProt:P26309 against the whole NR database. Boundaries of the WD domain and functional motifs, as defined in UniProt, are highlighted with green lines. **c** A zoom-in view of the heat map fragment centered on D-box. **d** A zoom-in view of the heat map fragment centered on C-box. **e** A zoom-in view of the heat map fragment showing the upper-left corner of the WD-repeats domain

Figure 3 demonstrates how CoeViz can facilitate identification of functional linear motifs and structural domains on the example of the anaphase promoting complex/cyclosome (APC/C) activator protein Cdc20 from baker’s yeast (SwissProt: CDC20_YEAST). It regulates the ubiquitin ligase activity and substrate specificity of APC/C (see UniProt:P26309 for references). According to UniProt annotation, Cdc20 comprises 7 WD structural repeats, and the following linear motifs: D-box (17-RSVLSIASP-25), bipartite nuclear localization signal (NLS, 85-RRDSSFFKDEFDAKKDK-101), C-box (144-DRYIPIL-150), and KEN-box (586-KENRSKN-592). As can be seen from the secondary structure (SS) prediction by SABLE [35], Cdc20 contains only one structural domain formed by WD repeats (Fig. 3a). Functional motifs are located in disordered (coil) regions of the protein, and therefore they would be obscure to the other, domain/family profile-oriented coevolution approaches, since the MSA would not cover those regions.

ProSite [36], one of the prominent resources for protein sequence annotations, finds only 4 WD repeats in the sequence and no motifs mentioned above. On the other hand, CoeViz with *MI* metric adjusted for phylogeny noise reveals boundaries of the WD-repeats domain and locations of D- and C-boxes (Fig. 3b-e). There have been observations published that short linear functional motifs are more conserved than their flanking (or adjacent) residues or the same motifs in non-functional instances (see review [37]). We suggest that coevolutionary information may amplify this signal because of the cooperative nature of these motifs, where more than one residue needs to be conserved to perform the function. However, this analysis is beyond the scope of this work.

## Conclusions

Coevolution analysis may facilitate the finding of groups of residues involved in the same function or domain folding. CoeViz both computes a number of coevolution and conservation metrics and provides interactive interface to analyze the data and identify relevant clusters of residues. The problem of potential phylogenetic bias in the MSA is addressed by a number of ways, including the use of the sequence databases with reduced redundancy, explicit phylogeny correction for similar sequences, and average product correction for mutual information. The tool represents a practical resource for a quick sequence-based protein annotation for molecular biologists, e.g., for identifying putative functional regions and structural domains. CoeViz also can serve computational biologists as a resource of coevolution matrices, e.g., for developing machine learning-based prediction models.

### Availability and requirements

- Project name: CoeViz
- Project home page: http://polyview.cchmc.org/
- Operating system: Platform independent
- Programming languages: Perl, JavaScript, R
- Other requirements: A web-browser supporting the HTML5 standard
- License: Free for all users
- Any restrictions to use by non-academics: None

## Abbreviations

APC: average product correction
DCA: direct coupling analysis
MSA: multiple sequence alignment
NCBI: national center for biotechnology information
NLS: nuclear localization signal
PDB: protein data bank
PSSM: position specific scoring matrix
SCA: statistical coupling analysis
SS: secondary structure
